# The establishment of a primary spine care practitioner and its benefits to health care reform in the United States

**DOI:** 10.1186/2045-709X-19-17

**Published:** 2011-07-21

**Authors:** Donald R Murphy, Brian D Justice, Ian C Paskowski, Stephen M Perle, Michael J Schneider

**Affiliations:** 1 Clinical Director, Rhode Island Spine Center, 600 Pawtucket Avenue, Pawtucket, RI 02860 USA; 2 Clinical Assistant Professor, Alpert Medical School of Brown University, Box G-A, Providence, RI 02912 USA; 3 Private Practice of Chiropractic, Rochester Chiropractic Group, 1687 English RoadRochester, NY 14616 USA; 4 Medical Director, Medical Back Pain Program at Jordan Hospital, 10 Cordage Park Circle, Suite 225, Plymouth, MA 02360 USA; 5 Professor of Clinical Sciences, University of Bridgeport, Bridgeport, CT 06604 USA; 6 Assistant Professor, School of Health and Rehabilitative Sciences, University of Pittsburgh, 4028 Forbes Tower, Pittsburgh, PA 15260 USA

**Keywords:** Low Back Pain, Neck Pain, Health Care Reform, Primary Care, Health Policy

## Abstract

It is widely recognized that the dramatic increase in health care costs in the United States has not led to a corresponding improvement in the health care experience of patients or the clinical outcomes of medical care. In no area of medicine is this more true than in the area of spine related disorders (SRDs). Costs of medical care for SRDs have skyrocketed in recent years. Despite this, there is no evidence of improvement in the quality of this care. In fact, disability related to SRDs is on the rise. We argue that one of the key solutions to this is for the health care system to have a group of practitioners who are trained to function as primary care practitioners for the spine. We explain the reasons we think a primary spine care practitioner would be beneficial to patients, the health care system and society, some of the obstacles that will need to be overcome in establishing a primary spine care specialty and the ways in which these obstacles can be overcome.

## Introduction

One of the most talked about issues in the United States (US) is health care reform. In other countries as well, discussion commonly revolves around the issue of how health care services can be improved while containing costs. Many in the US have described the current health care situation as a "crisis" [1-4]. In March 2010, the US Congress passed and the President signed into law the Affordable Care Act, which puts in place comprehensive health care reform measures [5]. While various models for providing care to patients have been considered, such as accountable care organizations [6], it is recognized that any meaningful approach to health care reform will require three goals to be achieved: 1. improved patient health; 2. improved patient experience; 3. decreased per capita costs [7].

Spine-related disorders (SRDs) are among the most common, costly and disabling problems in Western society. For the purpose of this commentary, we define SRDs as the group of conditions that include back pain, neck pain, many types of headache, radiculopathy, and other symptoms directly related to the spine. Virtually 100% of the population is affected by this group of disorders at some time in life. Low back pain (LBP) in the adult population is estimated to have a point prevalence of 28%-37%, a 1-year prevalence of 76% and a lifetime prevalence of 85% [8,9]. Up to 85% of these individuals seek care from some type of health professional [10,11]. Two-thirds of adults will experience neck pain some time in their lives, with 22% having neck pain at any given point in time [12].

The burden of SRDs on individuals and society is huge [13]. Direct costs in the United States (US) are US$102 billion annually [14] and $14 billion in lost wages were estimated for the years 2002-4 [13]. Other indirect costs are substantially higher than this. As far back as 1996 it was estimated that in The Netherlands total costs for neck pain was US$686 million, with half of that cost arising from disability [15]. And the problem appears to be worsening. In the years between 1997 and 2005, expenditures for back and neck pain rose 65%, adjusted for inflation [14]. During this time measures of mental health, physical functioning and work, school and social activity among patients with SRDs declined [14]. With regard to work-related LBP, this is the most common disorder that leads to lost work days [16] and while it comprises up to 25% of injuries in the workplace it accounts for up to 1/3 of all workers' compensation costs [17,18].

A variety of physicians and other providers have traditionally been involved with the diagnosis and treatment of these patients. This includes primary care physicians, chiropractic physicians, orthopedic surgeons, neurosurgeons, physiatrists, osteopathic physicians, physical therapists, psychologists, massage therapists, kinesiologists, naprapaths and acupuncturists. This has resulted in what has been termed the "supermarket approach" to the management of SRDs [19]. That is, the SRD patient is faced with an environment in which there is a large number of practitioners, each offering a solution to SRDs, with the patient left to sort out which of these disparate approaches is best for his or her particular problem. Oftentimes this determination is based more on salesmanship and marketing than on science, clinical benefit and cost-effectiveness [19].

Treatment for SRDs has become increasingly specialist-focused, imaging-oriented, invasive and expensive. According to Deyo, et al [20] between 1994 and 2004 LBP related Medicare expenditures in the US increased 629% for epidural steroid injections, 423% for opioid medications, 307% for magnetic resonance imaging and 220% for lumbar fusion surgeries. This dramatic rise in medical costs was not shown to have resulted in improved outcomes for SRD patients. In fact, despite the tremendous amount of time and money spent on the diagnosis and treatment of patients with SRDs, chronicity and disability related to these disorders appears to be steadily on the rise [14,20,21]. We are not aware of any other health condition in which a similar level of worsening outcomes has occurred despite significant increase in health care expenditures.

One approach to health care reform would designate primary care physicians (PCPs) or groups of PCPs as "patient homes", responsible for the comprehensive care and management of a designated patient population under a risk-sharing agreement. However, there is a projected gap between the availability of traditional PCPs and societal needs in the near future, especially if a national health care program is implemented [22]. Currently, LBP is the second most common reason for symptomatic physician visits [23-25] and increasing the number of SRD patients seeing PCPs will serve to further exacerbate the problem of under-availability of traditional PCPs. Thus, in the area of SRDs, a different approach to primary care is needed.

In their book Redefining Health Care [26], Porter and Teisberg state that for health care reform to be successful, it must incentivize competition based on *value*, i.e., outcome per dollar spent. To maximize value in health care, they recommend physicians and other health care providers organize themselves around conditions in which they have maximal expertise and experience (chronic kidney disease, diabetes, SRDs) rather than around medical specialties (orthopedics, internal medicine, neurology, etc.) and compete on the level of providing the best health outcomes for these conditions at the best possible cost (i.e., providing value). Having groups organized based on their medical specialty rather than their focused expertise is inefficient because different health conditions require different diagnostic strategies, treatment approaches, outcome measurements and monitoring [26].

SRDs have specific features that differentiate them from other types of health conditions. For example, diagnosis is challenging because, unlike conditions such as heart disease and diabetes, there usually is no well-defined lesion that can be clearly detected via imaging studies or other special tests [27]. In addition, many, and perhaps most, cases of SRDs are multifactorial, involving somatic, neurophysiological and psychological processes that interact to produce the suffering experienced by the patient [28,29]. Thus, management of patients with SRDs requires a level of expertise that can respond to these challenges.

In our view, a fundamental problem lies at the heart of the "supermarket approach" to SRDs; the lack of a "general practitioner" who has advanced training in spine care, who understands the multifactorial nature of SRDs and who can sort out the most appropriate clinical choices for the patient with low back or neck pain. Essentially, we think that the health care system needs an appropriately trained and skilled clinician who can fill the role of a primary care provider for the diagnosis and non-surgical management of SRDs; a "primary care physician for the spine".

### Primary Care for the Spine

"Primary care" is defined by the American Academy of Family Physicians (AAFP) as "that care provided by physicians specifically trained for and skilled in comprehensive first contact and continuing care for persons with any undiagnosed sign, symptom, or health concern (the "undifferentiated" patient) not limited by problem origin (biological, behavioral, or social), organ system, or diagnosis" [30]. The role of the traditional PCP is to apply comprehensive knowledge about the differential diagnosis of conditions that might arise in *any *bodily system, including the spine and musculoskeletal system. However, recent studies have shown that traditional PCPs are not well trained in the differential diagnosis and management of musculoskeletal disorders [31-33], probably due to the heavy emphasis on internal diseases in medical school education and in primary care residency programs. Even those traditional PCPs who profess to have a special interest in SRDs tend to have anachronistic beliefs about best practices for managing these disorders [34]. And guidelines do little to change practitioners' beliefs and practice [35]. The traditional PCP is not likely to be the best choice in the primary care of SRDs [36].

We are not using the term *primary care *in the context of a generalist who provides medical care for any condition involving virtually any organ system. We are using the term *primary spine care *in the context of a focused practitioner who provides medical care for all patients with problems related to a specific organ system - the spine. This model is analogous to the general dentist, who provides "primary care" for oral health. To paraphrase the AAFP definition for our purpose, "primary spine care" can be defined as "that care provided by practitioners specifically trained for and skilled in comprehensive first contact and continuing care for persons with any undiagnosed sign, symptom, or health concern (the "undifferentiated" patient) not limited by problem origin (biological, behavioral, or social), *involving the spine*".

Primary spine care would be provided by practitioners who are specifically trained to diagnose and manage the majority of patients with SRDs with the most evidence-based methods. They would also coordinate the referral and follow up of the minority of SRD patients who might require special tests (e.g. radiographs, MRI or electrodiagnostic testing) or more intensive (e.g. multidisciplinary rehabilitation) or invasive (e.g. injection and surgery) procedures.

The primary spine care practitioner would function as the first contact for patients with SRDs, i.e. the first practitioner that a patient consults when he or she develops a spine problem. The primary spine care practitioner could also function as a resource for traditional PCPs (family practice physicians, general internal medicine physicians, pediatric, obstetrical/ gynecological physicians, primary care nurse practitioners or physician's assistants) to refer patients who present with SRDs.

### The Necessary Skill Set of the Primary Spine Care Practitioner

The primary spine care practitioner would require several important characteristics in order to provide maximum value to society. Some of these characteristics include:

1*. Skills in Differential Diagnosis*: Serious pathology as a cause of spinal pain occurs in only 1% of patients [37]. However this means that the busy primary spine care practitioner could potentially see at least one case every couple of months. Thus, skill in the recognition of serious pathology is essential, as many of these disorders require immediate investigation or treatment. This includes an understanding of what diagnostic tests to order when certain "red flags" are present. Also essential in this regard is an understanding of when diagnostic testing is *not *necessary [38] as efficiency and cost-effectiveness would be an essential aspect of primary spine care.

2*. Skills in the management of the majority of patients with spine pain*: Any primary level practitioner should ideally be able to manage the majority of patients he or she sees without the need for referral. The first-line treatments that the primary spine care practitioner would employ would include those methods shown to be evidence-based, minimally invasive and cost-effective. There is a variety of such treatment methods that have been found to be effective and have broad application which include manual therapies, particularly manipulation and mobilization [39,40], the McKenzie method [41], neural mobilization techniques [42-44], various forms of exercise [45-47], patient-specific, evidence-based education [47,48], non-steroidal anti-inflammatory and non-opioid analgesics [27] (most of which are available over-the-counter) and nutritional approaches [49,50]. The primary spine care practitioner would be required to be knowledgeable and skilled in the application of these strategies without the need for referral.

3*. A wide ranging understanding of spinal pain*: SRDs are currently understood to be a complex mixture of biopsychosocial phenomena [29,51,52]. It is increasingly being recognized that the experience of spinal pain and its related disability involves a combination of biological and psychological processes that occur within a certain social context. The primary spine care practitioner would require a keen understanding of these disparate but interrelated processes. Patient satisfaction in spine care is closely tied to the clinician providing a clear explanation of the problem [53,54]. Therefore, the primary spine care practitioner would be required to clearly articulate the complexities of spine pain to patients in simple terms. The ability to recognize the many facets of some complex SRDs [28], educate the patient about his or her condition, its natural history and the patient's role in recovery [55], and then motivate the patient to actively participate in care [56] are all necessary, but quite refined, skills that the competent spine provider must have.

4*. The ability to detect and manage psychological factors: *It is increasing recognized the psychological factors play an important, and in many cases the most important, role in the perpetuation of pain, suffering and disability in patients with SRDs [57-60]. The primary spine care practitioner would have to be knowledgeable and skilled in the detection of processes such as fear-avoidance, catastrophizing, passive coping, poor self-efficacy, cognitive fusion and depression and to be able to address these as part of the overall management strategy [61]. As a purely psychological approach may not be effective [62] it is essential that management of these factors is incorporated by the primary spine care practitioner into the management of the somatic factors [63,64].

5*. An appreciation of minimalism in spine care*: The primary spine care practitioner would have to understand that often in spine care "less is more". That is, an approach that focuses on education regarding the natural history of SRDs, maximizes patient empowerment and minimizes practitioner-driven intervention is likely to be most beneficial [65,66]. This would allow the practitioner to focus on the *value *of care (i.e. outcome per unit cost [67]) which would not only benefit patients with SRDs but also the health care system and society as a whole by helping control costs while expediting early return to a productive life. This approach would also minimize the growing problem in spine care of patient dependency, whether on pharmaceuticals, interventional procedures, passive modalities or other practitioner-provided services [56].

6*. An understanding of the methods, techniques and indications of intensive rehabilitation, interventional treatments and surgical procedures*: It would be the responsibility of the primary spine care practitioner to coordinate the referral and follow up for the minority of patients who need secondary and tertiary level treatment. This would require knowledge and experience regarding the appropriate indications for these interventions, an ability to explain them to patients and an ability to follow up with these patients after the intervention to monitor the progress and outcome [68].

7*. An understanding of the unique features of work-related SRDs: *SRDs that begin in the workplace have particular features that differentiate them from those that are not perceived as work-related [69-71]. Many physicians, particularly traditional PCPs, are uncomfortable with work-related back pain and have misperceptions about the important role that early return to work and return to other normal activities plays in recovery [72-74]. The primary spine care practitioner would be required to understand the nuances of work-related SRDs and the unique aspects of management that are required to effectively care for this patient population [75].

8*. An understanding of the unique features of SRDs related to motor vehicle collisions: *Similar to work-related SRDs, those related to motor vehicle collisions (particularly whiplash associated disorders) have particular features that require specialized knowledge. The primary spine care practitioner would require an understanding of issues that are unique to this type of patient such as injury mechanisms [76,77], patterns of injury [78-80], risk factors for chronicity [81], medicolegal reporting and the delicate balance between the need for early, aggressive treatment [82] and the potential role this can play in chronicity [65,66].

9*. Public Health Perspective*: The primary spine care practitioner would require a broad perspective regarding how spine problems and spine care fits in the grander scheme of public health. For example, many of the health conditions that are the focus of public health education and promotion campaigns are associated with SRDs as complicating factors. These include: smoking, obesity, type II diabetes, lack of physical exercise, and mental health disorders. Public health campaigns regarding SRDs are in the early stages [83,84] and it can be expected that further public health efforts regarding this widespread set of problems will be undertaken [85] and will require input from primary-level practitioners with expertise in this area.

10*. The ability to coordinate the efforts of a variety of practitioners*: As we stated earlier, a high-quality primary spine care practitioner should be able to manage the majority of patients with SRDs without the need for referral. However, in those patients who require specialized services, the primary spine care practitioner would have to be skilled in the coordination of these services and in follow up to ensure that maximum benefit is derived.

11*. The ability to follow patients over the long term*: As SRDs typically take on a recurrent course [86,87] that is life-long [88] the primary spine care practitioner would have to be skilled in the long term follow up of patients to monitor recurrences, teach patients how to effectively interpret and self-manage the majority of these recurrences, and provide management of those recurrences for which self-management is not effective.

### The primary spine care practitioner: potential benefits for patients

Any patient benefits that may result from a focused management strategy with a well trained primary spine care practitioner would have to be investigated through a rigorous research effort. However, based on the current understanding of SRDs we would anticipate a number of such benefits. Some examples include:

1*. Faster recovery: *By providing targeted, evidence-based care the well-trained primary spine care practitioner would avoid unnecessary treatment, promote active care plans and patient empowerment and appropriately triage when necessary [89]. This can be expected to facilitate maximal outcomes in the shortest time.

2*. Cost savings: *The primary spine care practitioner could save patients considerable time and money both at the point of encounter and in the future by ordering diagnostic tests only when necessary, applying evidence-based treatments, avoiding unnecessary treatment and taking a "less is more" approach through education and motivation in self-directed care [27].

3*. Avoiding iatrogenic disability: *Judicious use of imaging and appropriate communication of findings may also help avoid the iatrogenic disability that can arise as a result of the medicalization of imaging findings that are of questionable clinical significance, such as "disc degeneration" [90]. Inappropriate communication of diagnostic test results can lead to unnecessary catastrophizing of benign spine pain that may result in prolonged disability [91] and unnecessary invasive procedures [92]. Having a primary spine care practitioner who understands when advanced imaging is necessary and when it is not necessary, and who can put into the proper perspective the findings of these tests, can help to reverse the costly imaging- and specialist-dominated culture that has developed in the area of SRDs.

4*. Increased productivity: *Encouragement to remain active, particularly with work-related SRDs and engaging in a targeted stay at work/ return to work strategy [93,94] would lessen the likelihood of work loss and its resultant economic hardship [95].

5*. Decreased likelihood of becoming a "chronic pain sufferer": *Appropriate care plans that focus on active care and patient empowerment are likely to help the patient avoid becoming a chronic pain sufferer [96]. The recognition of "yellow flags" of psychosocial involvement can lead to early intervention, before these factors lead patients down the path of prolonged disability [58,61].

6*. High patient satisfaction: *In the age of consumer-driven health care, the importance of the patient's overall experience of health care is of great importance [97]. Cost effective and clinically effective care provided by a practitioner who has good communication skills to educate, motivate and empower the patient will likely lead to high levels of satisfaction [54,98].

7*. Shared decision making: *The primary spine care practitioner would have a wide-ranging understanding of the various diagnostic and management strategies available to patients with SRDs and thus could provide information, resources and support in making decisions regarding their care.

8*. Focus on prevention: *While no program of prevention of future SRDs has been shown to be completely successful, it has been demonstrated that taking a preventative approach can help limit disability related to SRDs [82,99,100] and well as reduce the frequency of future episodes [101,102].

### The primary spine care practitioner: potential benefits to society

As with patient benefits, research would be required to determine any societal benefits that may result from the institution of a primary spine care practitioner. However we anticipate that there are many potential benefits to society of having a practitioner who is charged with providing primary care for patients with SRDs. Some examples include:

1.*Knowledgeable care coordinator: *A wide variety of practitioners is currently involved in the management of SRDs with little coordination of their efforts [19]. This leads to inefficiency and compromises value [26]. In our view it would be much more efficient and valuable to create teams of professionals with expertise in SRDs working together to provide efficient and effective patient care [26]. The primary spine care practitioner could play the role of "team captain" by organizing and supervising the work of the various disciplines that may be contributing to the management of any particular patient. This could be expected to improve outcomes by turning what is oftentimes a disjointed effort into a coordinated effort. It would also be likely to help control costs by having a single person in charge of monitoring a particular treatment to determine if it is bringing about meaningful improvement and should continue or is not bringing about meaningful improvement and should be altered or stopped.

2.*SRDs as a public health initiative: *Increased recognition is being given to the potential of a public health approach to SRDs [84,85]. The primary spine care practitioner can spearhead efforts in this area to facilitate and implement such public health campaigns as well as reinforce public health messages on an individual level with patients. Community-wide approaches to back pain have been successful in the past [84]. These programs involve a consistent evidence-based approach by primary contact providers coupled with community-wide education programs to inform the public on how to prevent disability related to SRDs and what to do if spine pain occurs. The success of these programs requires an understanding on the part of the primary spine care practitioner of the essential public health messages regarding SRDs. A community-wide public health initiative regarding SRDs has the potential to save millions of dollars and to prevent needless human suffering [84].

3.*Improved worker productivity: *SRDs trigger significant amounts of absenteeism [103] and "presenteeism" (the worker being present at the workplace but with significant losses in work productivity) [104,105]. The economic impact of these losses to a community is substantial. The establishment of a primary spine care practitioner could potentially lead to significant community-wide savings in both direct [14] and indirect [106] costs of SRDs.

4.*Less long term disability: *A significant portion of health care costs related to SRDs goes toward the management of chronic and recurrent conditions [17,107]. Appropriate initial evaluation and treatment can significantly reduce the number of acute pain patients who become chronic [82], and to reduce the cost of medical care, lost productivity and disability. A "culture of disability" can spread through a family or business or community, creating emotional and financial hardship for society [108]. Having a primary spine care practitioner who is skilled in disability management could potentially help reduce the risk of long term disability by acting at the early stages of a SRD episode [109,110].

### The primary spine care practitioner: potential benefits for the health care system

At present the delivery of health care to patients with SRDs follows the inefficient and expensive "supermarket approach" [19]. Having a primary spine care provider to manage patients with SRDs may benefit the health care system in a number of ways, including:

1*. Controlling costs: *The health care system in Western Society has been burdened with runaway costs. In no area is this more an issue than with SRDs [20]. By having a primary spine care practitioner who has the skills to manage the majority of patients with SRDs without the need for special tests or referral to specialists or other practitioners, a dramatic decrease in the cost of SRDs could be realized.

2*. Unburdening traditional PCPs: *The traditional PCP has the responsibility of managing the overall health needs of his or her patients. This includes, in many cases, multiple co-morbidities. The primary spine care practitioner would handle a significant portion of the traditional PCP's current case load, increasing the PCP's availability to the numerous other responsibilities of these practitioners. Thus, traditional PCPs would benefit by being relieved of the burden of caring for a large group of patient complaints for which they have little training [31-33]. This could also potentially result in a decrease in the projected PCP shortfall [22]. Having a primary spine care practitioner to whom traditional PCPs can refer patients with SRDs, or whom these patients can consult directly without having to see their PCP (a more efficient pathway), would remove from the already-overbooked schedule of traditional PCPs those conditions (SRDs) for which they have minimal training in diagnosis and management. This will allow them to focus on what they do best.

3*. More strategic specialist referrals: *Specialists who care for patients with SRDs would benefit for a similar reason as would traditional PCPs. Many patients with SRDs who see specialists such as orthopedic surgeons, neurosurgeons, interventional physiatrists or pain management physicians have no indications for surgery, injections or other invasive procedures. In addition, it has been found that in many cases these specialists do not have a keen understanding of the management of non-surgical SRDs [111]. This is likely because the bulk of the training of these specialists is focused on the application of interventional and surgical procedures in complex cases. By having all SRD patients see the primary spine care practitioner, who is trained to recognize those who require more invasive procedures, only those patients who need such procedures would be channeled to the surgical or interventional specialist. This would allow these specialist practitioners to focus their practice on doing what they do best - applying skilled surgical or interventional procedures.

4*. Disruptive innovation: *The establishment of clinicians who can provide primary spine care would represent a significant "disruptive innovation" [112] in health care. According to Christensen, et al [112] disruptive innovation is the process in which complex, expensive products and services are transformed into simple, affordable ones. Disruptive innovation in any industry occurs when a company, a group of individuals, or a profession comes along with new ideas and a new approach that leads to the transformation of the industry so that products and services become dramatically more affordable and accessible. This happened in the 1970s when Toyota disrupted the auto industry and in the early 1980s when Apple disrupted the computer industry [112]. We suggest that the introduction of the primary spine care practitioner can serve as a disruption in the delivery of spine care services that could potentially lead to dramatic improvements in the delivery, accessibility, cost and outcomes of this care. This viewpoint is supported by the example of the Spine Care Program at Jordan Hospital in Plymouth, Massachusetts where the primary spine care practitioner model has been implemented in an ACO-style environment. Preliminary evidence indicates that this program has been successful in the areas of outcomes, patient satisfaction and cost efficiency [113]. In addition, 80% of the patients in this program are referred by traditional PCPs supporting our viewpoint that the primary spine care practitioner model would be helpful in reducing the burden on these practitioners.

5*. Standardization of care: *Inconsistent clinical decision-making, unnecessary ordering of imaging studies, overutilization of invasive procedures, over-prescription of pharmaceuticals and excessive reliance on passive care approaches all trigger huge health care losses both in money and time [20]. A standardized, evidence based patient care pathway followed by knowledgeable practitioners has the potential to greatly minimize these costs.

6*. New evidence and technologies: *Currently, new treatment approaches or technologies regarding SRDs are often driven into the health care system more by marketing efforts than by good science [19]. With the introduction of a single group of primary spine care practitioners throughout the health care system, quality, evidence-based technologies and procedures could more quickly and efficiently be introduced.

### Obstacles to the implementation of the primary care for the spine model

There are a number of hurdles to overcome for the successful implementation of a primary care of spine model. These obstacles include:

1*. Educational changes: *Currently, none of the major health care educational institutions are consistently graduating providers who meet all the criteria necessary to be successful primary spine care practitioners. However with some basic fundamental changes, and a commitment from state and federal governments, trade organizations and school administrators and faculty, this obstacle can be overcome. Institutions of chiropractic medicine, for example, provide training that is focused primarily on the spine. Many of the skills required of the primary spine care practitioner are already taught at these schools. By instituting some specific changes, that are already being discussed within this health care profession [114,115], these institutions can become at least one source of appropriately trained primary spine care practitioners. Other disciplines that include some level of spine care training within their respective curricula are institutions of osteopathic medicine and physical therapy. The primary focus of most osteopathic programs in the US is the diagnosis and treatment of internal disorders with a majority of osteopathic physicians working in the field of family medicine. Physical therapy education does contain some spine related coursework, but is more broadly focused on musculoskeletal, neuromuscular, cardiopulmonary, and wound care. Thus, significant changes in these curricula would be required if they are to successfully train primary spine care practitioners.

2*. Incentivizing value: *Traditionally, in the area of SRDs and as in other areas of health care, providers have typically been paid by the procedure, thus incentivizing more procedures. This would have to change for successful implementation of primary spine care services into the health care system. Primary spine care practitioners would have to be adequately paid for activities such as patient education, coordination of care and stay at work/ return to work strategies. In addition, they would have to be financially incentivized to take a "less is more" approach. There are signs that this is starting to occur, however. As the health care system moves from fee for service toward a shared risk management model, providers and care pathways that add value to the system will be the leaders, thus increasing the support of their programs and services [67,97]. The concept of the primary spine care practitioner fits well into this model, allowing a "less is more" approach that involves fewer procedures and greater patient empowerment to replace the present "supermarket" approach [19] to SRDs.

3*. Overcoming prejudice: *It is likely that the best candidates to be groomed to become primary care spine providers may not come from the allopathic medical profession. This may be resisted in some aspects of the medical community. It would be important that a competent, appropriately trained provider be accepted regardless of the degree after his or her name. The institution of new models of health care in general, including primary spine care, will require non-traditional ways of thinking about which provider will become the "team captain" for any particular medical condition.

4*. The detrimental effect on those invested in the "supermarket approach": *For health care practitioners who currently see a large volume of patients with SRDs and who remain invested in the current incentive system in which more procedures are emphasized without regard for outcome or value, the institution of a primary spine care practitioner could be detrimental. If a system in which value rather than volume is rewarded, some practitioners will be negatively impacted and some may even go out of business [26]. Thus, the disruption of the health care system that the institution of a primary spine care practitioner will be a part of will undoubtedly be resisted by some individuals or groups who are unable or unwilling to embrace this change. However, such resistance has occurred in response to major disruptions of other industries [112] and we would anticipate that the benefits of the disruption we are suggesting will overcome any opposition that will inevitably arise.

5*. Resistance from within the profession(s) that could potentially be the source of primary spine care practitioners: *For whatever profession or professions that respond to the need for a primary spine care practitioner, this will be a significant disruption to the traditional practice patterns or self-image of these professions. As a result, the role that we are introducing here will be actively resisted [115]. However, given the fact that SRDs affect virtually 100% of the population it can be expected that whatever profession accepts the role of primary spine care practitioner will likely dramatically increase the volume of patients that seeks its services.

6*. Implementation: *The implementation of primary spine care services will require support from several areas of the health care system, including the profession(s) from which the non-surgical spine care practitioner will arise, third party payors, who will have to provide the financial incentive to bring value to spine care, regulatory and legislative bodies that may have to institute changes in allowing this area of health care to fully realize its societal benefits and other members of the health care system who will have to support and accept the implementation of primary spine care services. Again, disruptive innovations in other industries have required such changes and we would anticipate that the same can occur in response to the primary spine care innovation.

7*. Sustainability: *Any disruptive innovation has to be sustained in order for society to fully realize its benefits. Because of the great need we have presented here for high-quality, low cost (i.e., valuable) spine care, we feel that this need, and the benefits realized as a result of the institution of primary spine care services, will drive the sustainability of these services. However, this sustainability will also be dependent on the consistent supply of practitioners who are appropriately skilled in providing primary spine care. As we indicated earlier, this will require commitment on the part of whatever health care profession(s) elects to supply the system with appropriately trained practitioners.

## Conclusion

The need for some type of reform in our health care system is recognized by the public, industry and providers. The exact form that health care reform will take is not known but it is widely held that primary care services will have a significant clinical and administrative role and that shared risk among all stakeholders will be beneficial. Any meaningful approach to health care reform will require that three goals be achieved: 1. improved patient health, 2. improved patient experience 3. decreased per capita costs. That is, emphasis must be placed on value in health care. To achieve these goals, health care services in general must be redesigned away from the traditional fee-for-service model to a model based on value that is accessible, practical and sustainable.

It is our view that the addition of a primary spine care provider who is responsible for front-line diagnosis, management and triage would help achieve these goals, bringing greater value in the care of patients with SRDs. Moreover, the addition of this practitioner would be aligned with developing models of health care such as the patient-centered medical home and the accountable care organization. The establishment of such a practitioner is not unprecedented; primary oral health care is currently provided by the general dentist, who manages the majority of society's oral health needs him- or herself, with referral to specialist practitioners in those relatively few circumstances in which it is warranted. We think that the same model can be applied to SRDs.

The primary spine care practitioner will require a particular skill set that includes the ability to apply evidence-based procedures, appropriately educate and motivate patients and effectively prevent and manage disability related to SRDs. The benefits in terms of improved outcomes of care for SRDs, improved patient satisfaction, and reduced costs (i.e., the value of care for SRDs) would be well worth the effort of grooming practitioners toward filling this role.

## Disclosures

The authors declare that they have no competing interests.

## Authors' contributions

DRM originally conceived of the conceptual basis of the paper and wrote the initial manuscript. BDJ, ICP, SMP and MJS then made individual contributions to various sections of the manuscript. All authors took part in editing and revising the manuscript on multiple occasions. All authors reviewed the final manuscript prior to submission.
